# Adaptive designs in clinical trials

**DOI:** 10.4103/2229-3485.76286

**Published:** 2011

**Authors:** Suresh Bowalekar

**Affiliations:** *Managing Director, PharmaNet Clinical Services Pvt. Ltd., Mumbai, Maharashtra, India*

**Keywords:** Adaptive designs, Bayesian approach, interim analysis, randomization, sequential methods

## Abstract

In addition to the expensive and lengthy process of developing a new medicine, the attrition rate in clinical research was on the rise, resulting in stagnation in the development of new compounds. As a consequence to this, the US Food and Drug Administration released a critical path initiative document in 2004, highlighting the need for developing innovative trial designs. One of the innovations suggested the use of adaptive designs for clinical trials. Thus, post critical path initiative, there is a growing interest in using adaptive designs for the development of pharmaceutical products. Adaptive designs are expected to have great potential to reduce the number of patients and duration of trial and to have relatively less exposure to new drug. Adaptive designs are not new in the sense that the task of interim analysis (IA)/review of the accumulated data used in adaptive designs existed in the past too. However, such reviews/analyses of accumulated data were not necessarily planned at the stage of planning clinical trial and the methods used were not necessarily compliant with clinical trial process. The Bayesian approach commonly used in adaptive designs was developed by Thomas Bayes in the 18th century, about hundred years prior to the development of modern statistical methods by the father of modern statistics, Sir Ronald A. Fisher, but the complexity involved in Bayesian approach prevented its use in real life practice. The advances in the field of computer and information technology over the last three to four decades has changed the scenario and the Bayesian techniques are being used in adaptive designs in addition to other sequential methods used in IA. This paper attempts to describe the various adaptive designs in clinical trial and views of stakeholders about feasibility of using them, without going into mathematical complexities.

## INTRODUCTION

Since the introduction of the controlled clinical trials by Sir Austin Bradford Hill in 1946, the double-blind, randomized, controlled clinical trials have earned a status of a principal method for evaluation of new drugs or new therapeutic procedures in medicine. However, ethical and economic reasons created a need for sponsors and researchers to appraise or review the interim data at regular intervals for establishing or otherwise the efficacy and safety of medicines during the conduct of trial. Thus, periodic assessment of interim data and accordingly modification of the design of an experiment based on the analysis/review of accrued data has been a frequent and necessary practice for many years in drug development. In those days, the tendency was to adopt statistical procedures available in the literature and apply them directly to the design of the clinical trials. However, those procedures were not compliant with clinical trial practice, hence, were not accepted as best tools to handle certain situations. The option of early termination was not available in classical clinical trial designs, which advocated the use of only fixed sample-size trials. Repeated analysis of data from a classical or fixed sample-size trial results in inflating probabilities of type I error (also called as α or false positive) or type II error (called as β or false negative) above the predefined levels. This has motivated classical statisticians to develop statistical methods or tools and techniques to control the unwanted inflation of probabilities of errors. The outcome of these efforts was the development of sequential methods.

## INTERIM ANALYSES AND SEQUENTIAL METHODS

Interim analysis (IA), as defined in Food and Drug Administration (FDA)’s International Conference on Harmonization (ICH) guidance (ICH E9 Guidance), is “any analysis intended to compare treatment arms with respect to efficacy and safety”.

While performing IA, in the past, the statistical techniques got evolved, developed, refined and resulted in a set of relevant methods called as sequential methods.

The sequential methods that developed in a period of about past four decades or more are given below in brief:

### Classical open and closed sequential designs

Wald introduced Sequential Probability Ratio Test (SPRT) which is referred to as open sequential design.[5] This was improved by Armitage who proposed closed/restricted sequential designs.[6]

### Response adaptive and Bayesian approaches

Method by Noel, which proposed to adjust the treatment allocation sequentially based on patient response.[7]

### Group sequential methods

- *Ad hoc* rules: Haybittle[8] and Peto[9] used *ad hoc* rules not necessarily based on any precise theoretical model.
- Pocock[10] and O’Brien-Fleming boundaries.[11]
- Application to survival data.[12]
- α spending (use) function approach.[13]
- Pseudo sequential and semi-Bayesian approaches.

Thus, the sequential methods developed by Wald, Armitage and others have got tailored, over a period of time, to conform to the realities and logistical problems of clinical trial conduct. Sequential methods are now available to help better decision making regarding the benefit-to-risk ratio in a timely fashion.

Thus, over a long period of time, IA of accumulated data was carried out before the completion of study for the following five reasons:[14]

- Trends in aggregating safety data: Increased frequency of serious adverse events.
- Abandoning lost causes: Detect the compound which does not have the intended effect as early as possible in the study.
- Generation of new hypothesis: Unexpected findings of the present study suggesting future studies to test the new hypothesis.
- Resource and productive designs: To make resource allocation and project prioritization decisions as the trial progresses on the basis of IA of accumulated data.
- Overwhelming efficacy in life-threatening conditions: To stop trial early when the benefit is clearly outweighed by the risk of treatment.

There are also logistics issues encountered while using IA, which have been discussed in detail by E. W. Rockhold and G. G. Enas.[14]

IA of accumulated data was also performed in the past in the following situations:

- Stepwise, adaptive dose allocation in dose response studies;
- Early termination of a trial;
- Sample size re-estimation; and
- Play the winner – randomization.

## WHY ADAPTIVE TRIAL DESIGNS?

The white paper entitled “Critical Path Initiative” (CPI) was released by the US FDA in March 2004.[15] In this paper, FDA has shown a concern for the decline in the number of submissions for new drug approval. The need for innovative methods was strongly felt by regulators. IA was used by many researchers to get rid of the situation and bring down the time and cost of new drug development. As highlighted above, IA provided some kind of comfort to the researchers in making mid-term review of data and modifying the design accordingly. However, there were some problems encountered by statistical reviewers at FDA.[16] Some of them are listed below:

- Conducting one or more unplanned (*post hoc*) IA without any stated purpose or reason.
- Leaving the final *P* values unadjusted for interim analyses.
- Resizing the trial without *P* value adjustment after interim analyses on treatment differences.
- Changing the design and conduct of the trial after interim looks without addressing their impact on the final results.

Perhaps, due to this, one of the innovations which were forcefully recommended in the CPI was the use of adaptive design methods with use of Bayesian techniques in clinical trials to have inbuilt flexibility to get advantage of benefit of trial dugs without compromising the scientific integrity and validity. Adaptive design methods also have some teething troubles which are likely to slowly vanish with the increasing knowledge gained with its consistent use over a period of time.

## ADAPTIVE TRIAL DESIGNS

Adaptive clinical trials are also defined as “the studies that incorporate preplanned, mid course adjustments to study design based upon accumulating study data”.[17]

Thus, it is clear that in the adaptive clinical trial designs, the required changes are based on the review of accumulated data and are not made on an *ad hoc* basis. The changes are a part of “design” itself meaning adaptation is feature of adaptive design used to enhance the trial. It is not a remedial action to compensate for inadequate planning.[18]

Vladimir Dragalin[19] defines *adaptive design* as “a multistage study design that uses accumulating data to decide how to modify aspects of the study without undermining the validity and integrity of the trial”.

It is also explained that maintaining study

- *Validity* means providing correct statistical inference (such as adjusted *P* values, unbiased estimates, adjusted confidence intervals, etc.), ensuring consistency between different stages of the study, and minimizing operational bias and
- *Integrity* means providing convincing results to a broader scientific community; preplanning, as much as possible, based on intended adaptations; and maintaining the blind of IA results.

The terms “adaptive trial design” and “flexible trial design” are used synonymously although the draft guidance for industry on Adaptive Design Clinical Trials for Drugs and Biologics released in February 2010 uses the term “adaptive trial design”. The guidance defines “adaptive design clinical study” as “a study that includes a *prospectively planned* opportunity for modification of one or more specified aspects of the study design and hypothesis based on analysis of data (usually interim data) from subjects in the study”. It further says “analyses of the accumulating *study data* are performed at prospectively planned time points within study

- In a fully blinded manner or in an unblinded manner and
- With or without formal statistical hypothesis testing”.

### Adaptations employed in adaptive designs

The phrase “prospectively planned” means the adaptations are required to be planned before data get reviewed or examined in an unblinded manner by team members involved in planning the revision. The changes or modifications in the following items can be included in the prospectively written protocol:

- Eligibility criteria for
  - Subsequent study enrollment or
  - Subset selection
- Randomization procedure
- Treatment regimen for different treatment groups – examples
  - Dose level
  - Schedule
  - Duration
- Total sample size of the study including early termination, if any
- Concomitant treatments used
- Planned follow-up/patient evaluation schedule – examples
  - Number of follow-up visits or time points
  - Time of last patient visit/observation
  - Duration of patient participation
- Primary endpoint – examples
  - Which one out of many outcome assessments
  - Simple or composite endpoint
  - Simple or part of composite endpoint
- Secondary endpoint/(s) – selection, fixing of order of importance
- Analytical methods used to evaluate endpoints – examples
  - Covariates of final analysis
  - Statistical methods
  - Type I error control

If the study design aspect/(s) are changed/modified/revised on the basis of information/data from the source/the study other than that from the specific study under consideration, then the study is not considered as “adaptive study”. The accumulated data set has to be collected from the same study.

### Types of adaptive designs

Adaptive designs can be classified into various types on the basis of adaptations used and/or on the basis of adaption rules used.

The types of adaptive designs classified on the basis of adaptations used[21] are given in Table 1.

**Table 1:** Types of adaptive designs

Type of adaptive design	Description in brief
Adaptive randomization design	Allows alterations in the randomization schedule depending upon the varied or unequal probabilities of treatment assignment
Treatment-adaptive randomization	Dropping a treatment arm, adding a new treatment based on analysis of accumulated data at planned intervals
Response-adaptive randomization, also known as “Outcome-adaptive randomization”	Starts with fixed allocation ratio. Based on findings of analysis at predefined intervals, more subjects to be allocated to treatment with high response (e.g., Play-the-Winner model)
	Or change allocation when a fixed number of events has been observed in an arm (e.g., number of deaths)
	Breaking of blind introduces risk of bias
Covariate adaptive randomization, also known as “Dynamic randomization”	The probability of being assigned to a group varies in order to minimize “covariate imbalance”. In diseases where diagnostic factors are known to affect response or clinical outcome of treatment, it is desirable to achieve covariate balance of these prognostic factors
	Can it be called as “randomization”?
Group sequential design	Introduced in 1970 to have preplanned looks at the data to decide if trial could be stopped early either for efficacy or futility. Group Sequential Designs (GSDs) are in use in “3 + 3” phase I trial design for finding maximum tolerated dose (MTD)
Sample size re-estimation (SSR) design	SSR Design allows for sample size adjustment or re-estimation based on review and analysis of planned accumulated data. Blinded or unblended on the basis of variability, power, treatment effect size and reproducibility
Drop-the-loser design	Allows dropping of the inferior treatment groups, retain the control arm, add new arm
Adaptive dose finding design	Used in early phase of clinical development to establish minimum effective dose and maximum tolerable dose (MTD). Using GSD mentioned above or Continual Re-assessment Method (CRM)/Bayesian’s approach or a combination of the two. With Bayesian approach, the probability that drug is effective is updated on the arrival of new data
Biomarker-adaptive design	Allows for adaptation based on responses of biomarkers. It is used to select the right patient population, find natural course of disease, early detection of disease;[22] in short, optimal screening, establish a validate predictive model
Adaptive treatment-switching design	Allows investigator to switch patient’s treatment from that initially assigned to alternative treatment. This is based on the evidence of efficacy or safety observed at review of accumulated data at preplanned intervals
Hypothesis-adaptive design	Allows change in hypothesis initially set to the other based on review of accumulated data. Examples are change from superiority to non-inferiority hypothesis, change of study endpoints. All these prior to data un-blinding and database lock
Adaptive seamless phase II/III trial design	It combines two trials – phase II (a) and phase III. Uses data on patients enrolled before and after adaptation for performing final statistical analysis
Multiple adaptive design	It is a combination of two or more of the above adaptations

## REGULATORY VIEW AND FUTURE DIRECTIONS

Adaptive/flexible design is a novel tool and needs to be used initially by appropriate scientific interactions with regulators, academicians, and statisticians to arrive at an optimal level of adaptations. “Too much of adaptations should be avoided in phase II confirmatory trials”.[23]

Hung *et al*.[23] have listed limited experiences as regulators under the following heads:

### Extension of maximum and total statistical information

It is suggested that a number of questions, at the design stage, when a protocol is designed to allow change of statistical information, should be asked in order to justify the design adaptation. In brief, the attempt should be to provide answer to the question “Is there sufficient justification to use these designs and where do we use them”.

### Drop a treatment/dose arm

Dropping a dose arm with lack of intended therapeutic effect or substantial side effect is frequent consideration in a number of regulatory submissions. Their experiences suggest avoiding reallocation of unused alpha in a trial that allows dropping of treatment arm at a mid study time.

The other heads discussed in detail by James Hung *et al*.[23] include

- Changing from superiority to non-inferiority and
- Changing primary endpoint.

The article by Hung *et al*.[23] also gives a good account of “issues with Statistical Methodology for Adaptive or Flexible Designs” with four case studies, in addition to logical issues, regulatory evaluation issues and infrastructure issues.

Mucke[24] predicts that “increasing guidance and endorsement from regulatory bodies will lead the industry to fully embrace adaptive trials by 2015”. The results of a survey conducted to illuminate current practices, plans and views regarding adaptive designs for clinical trials are spelt out in the paper by Mucke. The majority of respondents reported the following as the key points in favor of adaptive designs:

- Reduction in patient numbers,
- Less exposure to study drug and
- Decline in overall trial duration.

At the same time, there were concerns raised by most of the responders on methodological, logistical, and regulatory uncertainties. The methodological concerns are about the likelihood of reaching erroneous conclusions in case the adaptive and/“seamless” designs are used in phase II and pivotal studies. Whether such a trial could be kept fully under control without major organizational change/expansion and increased dependency on outside statistical or monitoring advice was the major concern under logistical concerns. The regulatory concern by the majority was about acceptance of adaptive designs and more importantly of the interpretations of results of such trials by the trial sponsors by regulatory authorities. The paper summarizes the following as the key challenges involved in adaptive trials:

- Staff training;
- Electronic data capture (EDC) to enable near-real-time capture, validation, and analysis of trial data;
- Working with Data Monitoring Committees (DMCs);
- Ways in which modifications like dropping and replacing a dosage arm can have ripple effects on a project’s critical path and
- The challenges of prognosis, analysis and interpretations.

In spite of all the issues and concerns, the best way to get appropriate solutions to the listed issues and to deal with various concerns will be to start using the adaptive trial designs with appropriate involvement of all stakeholders, right at the planning stage, where the planning should consider the entire development program as a whole. According to Hung *et al*.,[23] “benefits and drawbacks of adaptive or flexible designs over the traditional non-adaptive designs need to be assessed in real practical applications”.

## References

[1] Armitage P, McPherson CK, Rowe BC (1969). Repeated significance tests on accumulating data. Journal of the Royal Statistical Society Series A.

[2] Armitage P, Stratton IM, Worthington HV (1985). Repeated significance tests for clinical trials with a fixed number of patients and variable follow-up. Biometrics.

[3] Berry DA (1989). Monitoring accumulating data in a clinical trial. Biometrics.

[4] McPherson CK (1982). On choosing the number of interim analyses in clinical trials. Stat Med.

[5] Wald A (1947). Sequential Analysis.

[6] Armitage P (1969). Sequential analysis in therapeutic trials. Annu Rev Med.

[7] Noel DG, Sobel M, Weiss GH, Elashoff RM (1975). A survey of adaptive sampling for clinical trials. Perspectives of Biometrics.

[8] Haybittle JL (1971). Repeated assessment of results in clinical trials of cancer treatment. Br J Radiol.

[9] Peto R, Pike MC, Armitage P, Breslow NE, Cox DR, Howard SV (1976). Design and analysis of randomized clinical trials requiring prolonged observation of each patient. I. Introduction and design. Br J Cancer.

[10] Pocock SJ (1977). Group sequential methods in the design and analysis of clinical trials. Biomerika.

[11] O’Brien PC, Fleming TR (1979). A multiple testing procedure for clinical trials. Biometrics.

[12] Sooriyarachchi MR, Whitehead J (1998). A method for sequential analysis of survival data with nonproportional hazards. Biometrics.

[13] Lan KK, DeMets DL (1983). Discrete sequential boundaries for clinical trials. Biometrika.

[14] Rockhold FW, Enas GG, Peace KE (1992). Practical approaches to the design and conduct of interim analyses. Biopharmaceutical Sequential Statistical Applications.

[15] (2004). http://www.fda.gov/oc/initiatives/critical-path/whitepaper.html.

[16] Sankoh AJ (1999). Interim Analysis: an update of an FDA reviewer’s experience and perspective. Drug Inf J.

[17] Byrom B, McEntegart D (2009). Data Without Doubt GCPj 2009 (April); 12-15 via. http://www.perceptive.com/Library/Papers/Data_Without_Doubt.pdf.

[18] Gallo P, Chuang-Stein C, Dragalin V, Gaydos B, Krams M, Pinheiro J (2006). Adaptive designs in clinical drug development--an Executive Summary of the PhRMA Working Group. J Biopharm Stat.

[19] Dragalin V (2006). Adaptive Designs: Terminology and Classification. Drug Inf J.

[20] http://www.fda.gov/downloads/Drugs/guidancecomplianceregulatoryinformation/guidances/ucm201790.pdf.

[21] Chow SC, Chang M (2008). Adaptive design methods in clinical trials - a review. Orphanet J Rare Dis.

[22] Wang SJ, O’Neill RT, Hung HM (2007). Approaches to evaluation of treatment effect in randomized clinical trials with genomic subset. Pharm Stat.

[23] Hung HM, O’Neill RT, Wang SJ, Lawrence J (2006). A regulatory view on adaptive/flexible clinical trial design. Biom J.

[24] http://www.insightpharmareports.com/reports/2007/86_Adaptive_Clinical_Trials/overview.asp.

